# Metformin Alters the Chemotaxis and Flagellar Motility of *Escherichia coli*

**DOI:** 10.3389/fmicb.2021.792406

**Published:** 2022-01-11

**Authors:** Yingxiang Ye, Panmei Jiang, Chengyun Huang, Jingyun Li, Juan Chen, Lu Wang, Yan Lin, Fangbin Wang, Jian Liu

**Affiliations:** School of Food and Biological Engineering, Hefei University of Technology, Hefei, China

**Keywords:** *Escherichia coli*, metformin, flagellar motor, chemoreceptors, chemotactic response, motor torque, swimming motility

## Abstract

Metformin is a biguanide molecule that is widely prescribed to treat type 2 diabetes and metabolic syndrome. Although it is known that metformin promotes the lifespan by altering intestinal microorganism metabolism, how metformin influences and alters the physiological behavior of microorganisms remains unclear. Here we studied the effect of metformin on the behavior alterations of the model organism *Escherichia coli* (*E. coli*), including changes in chemotaxis and flagellar motility that plays an important role in bacterial life. It was found that metformin was sensed as a repellent to *E. coli* by tsr chemoreceptors. Moreover, we investigated the chemotactic response of *E. coli* cultured with metformin to two typical attractants, glucose and α-methyl-DL-aspartate (MeAsp), finding that metformin prolonged the chemotactic recovery time to the attractants, followed by the recovery time increasing with the concentration of stimulus. Metformin also inhibited the flagellar motility of *E. coli* including the flagellar motor rotation and cell swimming. The inhibition was due to the reduction of torque generated by the flagellar motor. Our discovery that metformin alters the behavior of chemotaxis and flagellar motility of *E. coli* could provide potential implications for the effect of metformin on other microorganisms.

## Introduction

Metformin is the most common prescribed drug in the treatment of Type 2 diabetes and other metabolic syndromes (Forslund et al., 2015; Wu et al., 2017; Fang et al., 2018). It has been reported that metformin has the potential benefits in reducing the risk of cancer and promoting lifespan in mammalians and nematode *Caenorhabditis elegans* (Cabreiro et al., 2013; De Haes et al., 2014). In addition, recent research demonstrated that metformin could modify the gut microorganism composition of diabetic patients treated with this molecule (Fang et al., 2018). Moreover, it was found that metformin alters folate and methionine metabolism of the gut bacterium *Escherichia coli*, which was the only single microbe that exists in the gut of *C. elegans*, to promote the lifespan of *C. elegans* (Cabreiro et al., 2013). Metformin was also found functioned in the adverse gastrointestinal effect involved in the genus *Escherichia* (McCreight et al., 2016). These findings reveal that metformin affects the intestinal microorganisms.

As a common bacterium in the intestine and environment, *Escherichia coli* can sense and respond to the extracellular gradients of chemicals to migrate toward favorable conditions *via* the chemotaxis system, with receptor clusters processing the input signal, while the flagellar motor generates output to drive the bacteria moving in a random walk pattern, alternating between runs and tumbles (Bray et al., 1998; Cluzel et al., 2000; Berg, 2003). The bacterium runs when all of the flagellar motors on the cell rotate in a counterclockwise (CCW) direction and reversely tumbles when one or more motors rotate in a clockwise (CW) direction (Turner et al., 2000). The bacterium performs a biased random walk that directs net migration in the presence of a stimulus gradient, which is regulated by the level of phosphorylated CheY (CheY-P) binding to the component of the flagellar motor–switch complex that controls the probability of the flagellar motor rotating in the CW direction (the CW bias). Exposure to attractants causes less CheY-P to bind to FliM, a component of the switch complex at the base of the flagellar motor, leading to a decrease in CW bias, whereas removal of the attractant causes more CheY-P to bind to FliM, thus increasing the CW bias (Sourjik and Berg, 2002). The chemotaxis system is a perfect robust system that could adapt to chemical gradients (Alon et al., 1999).

The flagellar motor rotation is pumped by the proton motive force (PMF) that drives the flow of protons through the inner transmembrane channels of the stator complex composed of the proteins MotA and MotB (Gabel and Berg, 2003; Kojima and Blair, 2004). PMF consists of a transmembrane voltage and a concentration difference across the membrane, both of which are maintained by various metabolic processes (Xing et al., 2006). The stator complex interacts with the rotor complex which consists of FliG, FliM, and FliN to generate the motor torque through electrostatic interactions (Minamino and Imada, 2015). The motor was shown to be a dynamic structure with stators binding on and off it. Interactions of phosphorylated CheY promotes the motor switches from the CCW direction to CW direction (Wang et al., 2017). The flagellar motility of the bacteria characterized by the bacterial swimming speed depends strongly on the rotation speed of the flagellar motor, which is controlled by the flagellar motor torque (Wadhwa et al., 2019; Namba, 2020). The chemotaxis and flagellar motility are important to bacteria such as *Escherichia coli*, which could affect a variety of key physiological functions of bacteria, including formation of microbial biofilm, changes in cell morphology, generation of pathogenic factors, and toxicity (Ophir and Gutnick, 1994; Hecht and Newton, 1995; O’Toole et al., 2000; Josenhans and Suerbaum, 2002; Butler and Camilli, 2004). In the case of pathogenic bacteria, chemotaxis and flagellar motility are crucial in the process of adhesion and invasion of the host, such as the adhesion of *Helicobacter pylori* to the mucus layer of the stomach (Pittman et al., 2001), and *Vibrio anguillarum* adheres to the surface of fish (O’Toole et al., 1999).

Previous study indicated that metformin could influence the metabolism of microorganisms. However, the effect of metformin on the behavior of bacteria remains unclear, such as chemotaxis and flagellar motility. In this study, we explored the behavior alterations of the model microorganism *Escherichia coli* affected by metformin, focusing on the chemotaxis and flagellar motility. We investigated the chemotactic response of *E. coli* to metformin and the effect of metformin on the chemotaxis of *E. coli* to attractant including glucose and MeAsp. We also systematically studied the effect of metformin on the motility of *E. coli* from the level of a single flagellar motor to individual cell and cell population.

## Materials and Methods

### Strains and Plasmids

Strains and plasmids are listed in Table 1. HCB33, JY26 (Δ*fliC*), and HCB269 (Δ*tsr*) are derivatives of *E. coli* K12 strain RP437. The plasmid pKAF131 expresses the sticky filament FliC*^st^* constitutively. The plasmid pFD313 also expresses the sticky filament FliC*^st^* constitutively. The plasmid pJY7 expresses wild-type MotA and MotB under the control of an arabinose-inducible promoter in the vector pBAD33. To measure the chemotaxis and motility for flagellar motor response to metformin, JY26 transformed with pKAF131 and HCB269 transformed with pKAF131 were used. To measure motility for flagellar motors with overexpressed MotA and MotB, JY26 transformed with pFD313 and pJY7 and HCB33 transformed with pJY7 were used.

**TABLE 1:** Strains and plasmids used in this experiment.

Strains	Relevant genotype	Source
HCB33	Wild-type	RP437
JY26	Δ*fliC*	RP437
HCB269	Δ*tsr*	RP437

**Plasmids**	**Relevant genotype**	

pKAF131	*fliC* *^st^*	
pFD313	*fliC* *^st^*	
pJY7	*motA motB*	

### Cell Culture

Cells of JY26 and HCB269 transformed with pKAF131 were grown at 33°C in T-broth with the appropriate antibiotics (25 μg ml^–1^ chloramphenicol). Cells of JY26 transformed with pFD313 and pJY7 were grown at 33°C in T-broth with the appropriate antibiotics (25 μg ml^–1^ chloramphenicol, 100 μg ml^–1^ ampicillin) and 0.01% arabinose. All of the strains cultured with the following concentrations (0, 5, 10, and 20 mM) of metformin were grown to OD_600_ between 0.45 and 0.50. A volume of 1 ml of the cells was collected by centrifugation at 5,000 × *g* for 1 min, washed twice in 1 ml of motility medium [10 mM potassium phosphate, 0.1 mM ethylenediaminetetraacetic acid (EDTA), 10 mM lactic acid, and 70 mM NaCl at pH 7.0], and suspended in 1 ml of this medium. The suspension was used for experiments immediately or stored at 4°C for up to 1 h. Cells of HCB33 transformed with pJY7 were grown at 33°C in T-broth with the appropriate antibiotics (25 μg ml^–1^ chloramphenicol) and 0.01% arabinose.

### The Bead Assay

For the bead assay, cells were sheared to truncate flagella by passing 1 ml of the washed cell suspension 50 times between two syringes equipped with 23-gauge needles and connected by a 7-cm length of polyethylene tubing (0.58 mm i.d., no. 427411; Becton Dickinson, Franklin Lakes, NJ, United States) and condensed into 300 μl of motility medium. Coverslips were covered by poly-L-lysine (0.01%, P4707; Sigma, St. Louis, MO, United States), and a chamber was formed with two pieces of double-sided tape spaced between the coverslip (20 mm × 20 mm) and a glass slide (75 mm × 25 mm). To measure the CCW and CW rotation velocities of the flagellar motor, 40 μl of cells was placed on the glass coverslip covered with poly-L-lysine and allowed to maintain for 3 min, syringed with 100 μl motility medium; then 1-μm-diameter polystyrene latex beads (0.13%, no. 07310-15; Polysciences, Warrington, PA, United States) were attached to the sheared flagellar stubs, incubated for 3 min, and rinsed with 100 μl motility medium again. The chamber was sealed with vacuum grease. Then, the polystyrene beads were observed by a Nikon Ti-2 phase-contrast microscope with a 40× objective and recorded with a scientific CMOS camera (DCC3260 M) at 500 frames per second. All experiments were carried out at 23°C. Data analysis was carried out using custom scripts in MATLAB.

### Microfluidics

For microfluidics, to perform long-time measurements of the chemotaxis, a flow chamber was constructed by using a rectangle-shaped double-sided sticky tape (about 100 mm thick) as a spacer between a glass coverslip (50 mm × 24 mm) coating poly-L-lysine and a glass slide (75 mm × 25 mm) with two 0.75-mm-diameter holes. The periphery of the chamber was sealed with Apiezon vacuum grease additionally. The flow chamber was kept under the constant flow of fresh motility medium at the rate of 250 μl/min using a syringe pump. The cells were added and incubated for 3 min. Then, a 0.13% (w/v) solution of 1.0-mm-diameter polystyrene beads was added and incubated for 3 min until enough beads were attached to the sheared flagellar stubs. Finally, the unstuck cells and beads were rinsed with the flow of motility medium. The rotation of the beads was monitored using a Nikon Ti-2 phase-contrast microscope with a 40× objective and recorded with a scientific CMOS camera (DCC3260 M) at 500 frames per second. All experiments were carried out at 23°C. Data analysis was carried out using custom scripts in MATLAB.

### 2D-Swimming Tracking and Trajectory Analysis

A volume of 1 ml of cells grown to an OD_600_ between 0.45 and 0.50 were harvested (1,200 × *g*, 6 min) and washed twice in fresh media, and cells were resuspended in 1 ml of 0.05% (wt/vol) PVP-40 in motility medium (to prevent cells from attaching to the glass). Resuspended cells were diluted to an OD_600_ of approximately 0.05. A volume of 10 μl washed bacteria was injected into glass chambers made up of coverslip and slide. The trajectories of swimming cells were observed by a Nikon Ti-2 microscope under a 10× objective, and videos were recorded with a CMOS camera (DCC3260 M) at 15 fps and exposure time of 50 ms. The frames were recorded for 100 s with the resolution of the frames of 300 by 300 pixels. ImageJ was used to analyze the trajectory of single bacteria and derive the information of all trajectories of each bacterial motion, and then data analysis was carried out using custom scripts in MATLAB. An example of bacterial trajectories is shown in Supplementary Figure 2. Bacterial trajectories of less than 5 s were discarded. Behavioral parameters such as swimming speed were extracted from single-cell trajectories. Gaussian fitting was carried out with MATLAB to obtain the average value of swimming speed.

### Dark-Field Flicker Microscopy

Coverslips and slides were thoroughly cleaned, and a chamber was formed with two pieces of double-sided tape spaced between the coverslip (20 mm × 20 mm) and a glass slide (75 mm × 25 mm). To measure the average head and flagella rotation velocities of the population of cells, 40 μl of cells was added to the chamber. The chamber was sealed with vacuum grease. The motion was observed using a phase-contrast Nikon Ti-2 inverted microscope with a 10× objective and a scientific CMOS camera (Flare 2M360-CL) at 500 frames per second with incident light provided by a 200-mW LED lamp immediately for a dark-field flicker mode of illumination. Video clips at a resolution of 512 by 512 pixels were recorded for approximately 20 s over an area of 332 μm^2^ by 332 μm containing around 500 cells. All experiments were carried out at 23°C. Data analysis was carried out using custom scripts in MATLAB.

### Speed and Torque of the Bacterial Flagellar Motors

Data analysis was done using custom scripts in MATLAB. Rotational speed was extracted as the peak of the speed histogram for each 200-s measurement. Relative torque was computed as speed times the rotational frictional drag coefficient of the 1.0-mm-diameter polystyrene beads. The torque was *T* = 2πωf_b_, where ω is the rotational speed of the flagellar motor and f_b_ is the drag coefficient of the beads with f_b_ = 8πηa^3^ + 6πηal^2^, where η is the viscosity of the medium and a and l are the radius of the bead and the distance between the rotation axis and the center of the bead (the major radius of the trajectory), respectively. a is 0.5 μm, l is about 0.20 μm, leading to f_b_ = 9πηa^3^. For each concentration of metformin, the means and standard error of the mean were computed for the cell ensemble, with each measurement weighted equally.

### Soft Agar Swimming Assays

Semi-solid motility plates were prepared using tryptone broth (13 g tryptone and 7 g NaCl per liter of media) and 0.25% agar and metformin. Plates were spotted with 4 μl of cell culture grown to OD_600_ between 0.45 and 0.50 in 10 ml T-broth. Plates were incubated at 37°C for 8 h and imaged on a Gel Imaging System. Culture diameters were measured using ImageJ software and normalized to the diameter of the petri dishes.

### Simulations

The attractant gradient was set up by the formula $G\,\left(x,y\right)=100\,\sqrt{x^{2}+y^{2}}$. Each cell transits stochastically between run and tumble; the transition rate K between run and tumble is set to be a constant for cells cultured with or without metformin. The locomotion speed is set to be 1 and 0.7 μm/s corresponding to the cells cultured with and without metformin. The simulation is updated every 0.1 s with the position recorded. Each cell was simulated for 100 s, and 100 cells were simulated for each type of cells.

### Statistics

In this study, GraphPad Prism 5 and Excel were used to determine the average values and standard errors. All the error bars represent the SEM. For each figure, the number of replicates and other information relevant for assessing the accuracy and precision of the measurements are included in the corresponding legend. As software tools, MATLAB, ImageJ, and OriginPro 8.5 were used.

### Data Availability

Data generated and analyzed during this study are available from the corresponding authors upon request.

## Results

### Metformin Is Sensed by tsr Chemoreceptors as a Repellent to *E. coli*

**FIGURE 1:**
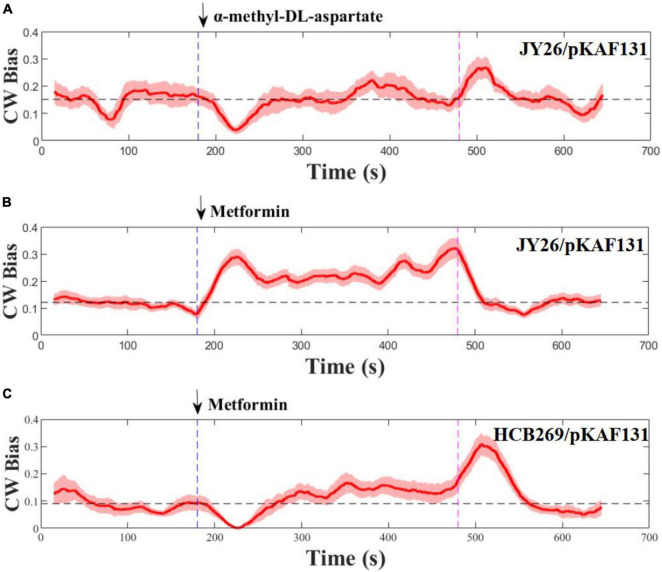
Flagellar motor response to stepwise addition and removal of α-methyl-DL-aspartate and metformin. The metformin was added (blue) and removed (purple) at the times indicated by the vertical dashed line. The dashed black line represents the average of the CW bias from 0 to 180 s. Error areas for standard errors of the mean are shown under the CW bias curve (light red). **(A,B)** Wild-type strains for chemotaxis, **(C)** is the strain (Δ*tsr*). The numbers of the flagellar motor measured at each section were 10, 28, and 12, respectively.

To test the chemotactic response of *E. coli* to metformin, the strain JY26 that was wild-type for chemotaxis was used for this experiment. We studied its response to a stepwise stimulus of 20 mM metformin, considering the growth inhibition of metformin to *E. coli* in different concentrations (Supplementary Figure 1). The flagellar motor CW bias (the probability of the flagellar motor rotating clockwise) was used as the indicator of chemotactic output (Min et al., 2012; Zhang et al., 2018). The flagellar motor rotation was measured for 11 min, divided by three phases: pre-stimulus phase (0–3 min), stimulus phase (3–8 min), and stimulus removal phase (8–11 min). The rotation traces of 28 motors on different cells were recorded and population-averaged to smooth over the variability of individual cells. MeAsp, a common strong attractant to *E. coli*, was used as the control (Mello and Tu, 2007). As shown in Figures 1A,B, the chemotactic response tendency of *E. coli* to metformin was in contrast with MeAsp, suggesting that metformin was a repellent to *E. coli*. The motor CW bias was around 0.12 before the addition of metformin. In contrast, the motor CW bias abruptly increased to near 0.3 after the addition of 20 mM metformin, then the motor CW bias gradually decreased and was maintained at around 0.21 which was higher than the pre-stimulus level in 60 s, consistent with the adaptation time measured at the level of the flagellar motor which was tens of seconds (Park et al., 2010). After removal of metformin, the motor CW bias decreased to the pre-stimulus level rapidly. The chemotaxis system of *E. coli* is a robust adaptive system which allows the bacteria to sense and respond rapidly to the changes in the environment (Alon et al., 1999; Liu G. et al., 2020). The motor CW bias was recovered to the initial level after rapid adaptation to the external attractant or repellent. However, the motor CW bias was adapted and maintained above the pre-stimulus level after the addition of metformin. Thus, metformin not only is a repellent to *E. coli* but also has a strong effect on the CW bias of *E. coli* evidently.

Previous study revealed that tsr chemoreceptors played a key role in sensing certain repellents (Callahan et al., 1987). To determine whether metformin is sensed by tsr chemoreceptors as a repellent, the strain HCB269 that is the knockout of *tsr* was used in the experiment. We measured its response to the stepwise stimulus of metformin, and the measurements are shown in Figure 1C. Interestingly, metformin was sensed by the cells as an attractant, in contrast to the result of the strain JY26. Therefore, the results above suggested that metformin was sensed by tsr chemoreceptors as a repellent to *E. coli*.

### Metformin Prolongs the Chemotactic Recovery Time of *E. coli* Responded to Attractant

**FIGURE 2:**
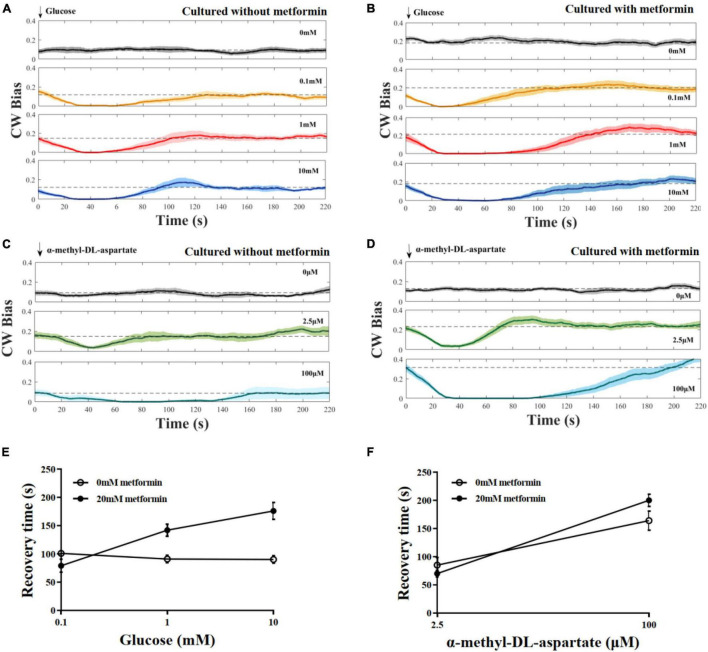
The chemotactic response of the flagellar motor of the wild-type strain for chemotaxis to stepwise addition of the attractant. **(A)** Cells were cultured in T-broth with 0 mM metformin. Glucose was added at *t* = 0. The numbers of different cells (each cell was stimulated only once) at each stimulus level: *n* = 12, 10, 10, and 10, from top to bottom. The recovery times corresponding to the glucose of different concentrations were 101 ± 4, 91 ± 6, and 90 ± 7 s, respectively (mean ± SEM). **(B)** Cells were cultured in T-broth with 20 mM metformin. The numbers of different cells at each stimulus level: *n* = 12, 11, 10, and 11, from top to bottom. The recovery times corresponding to the glucose of different concentrations were 79 ± 11, 142 ± 10, and 176 ± 15 s, respectively. **(C)** Cells were cultured in T-broth with 0 mM metformin. α-Methyl-DL-aspartate was added at *t* = 0. The numbers of different cells at each stimulus level: *n* = 10, 10, and 9, from top to bottom. The recovery times corresponding to the α-methyl-DL-aspartate of different concentrations were 85 ± 13 and 164 ± 17 s, respectively. **(D)** Cells were cultured in T-broth with 20 mM metformin. The numbers of different cells at each stimulus level: *n* = 12, 12, and 13, from top to bottom. The recovery times corresponding to the α-methyl-DL-aspartate of different concentrations were 70 ± 6 and 200 ± 11 s, respectively. **(E)** The average recovery time as a function of the concentrations of glucose (0.1, 1, 10 mM). Recovery time of cells cultured with 0 mM metformin (open black circles) and cultured with 20 mM metformin (solid black circles). **(F)** The average recovery time as a function of the concentrations of α-methyl-DL-aspartate (2.5 and 100 μM). Recovery time of cells cultured with 0 mM metformin (open black circles) and cultured with 20 mM metformin (solid black circles).

To investigate the effect of metformin on the chemotaxis of *E. coli* to the attractant, we measured the CW bias of the strain JY26. The cells were cultured with 20 mM metformin. As control, the CW bias of cells cultured without metformin was also measured. Glucose and MeAsp were used as the typical attractant in this experiment. The recovery time τ was defined as the duration time from the time the stimulus was added to the time CW bias returned back to its pre-stimulus level. Cells cultured with or without metformin showed diverse chemotactic responses to the attractant and various recovery times τ, although the tendency of chemotactic response was similar (Figures 2A–D). Interestingly, the recovery time of cells cultured with metformin was increased with the concentration of glucose, in contrast with the result that the recovery time of cells in control was unchanged with the concentration of glucose (Figure 2E). The recovery time of cells to MeAsp was also increased with the concentration of metformin (Figure 2F). Hence, the results above indicate that metformin prolonged the recovery time of *E. coli* chemotactic response to the attractant.

### Metformin Inhibits the Flagellar Motility of *E. coli*

**FIGURE 3:**
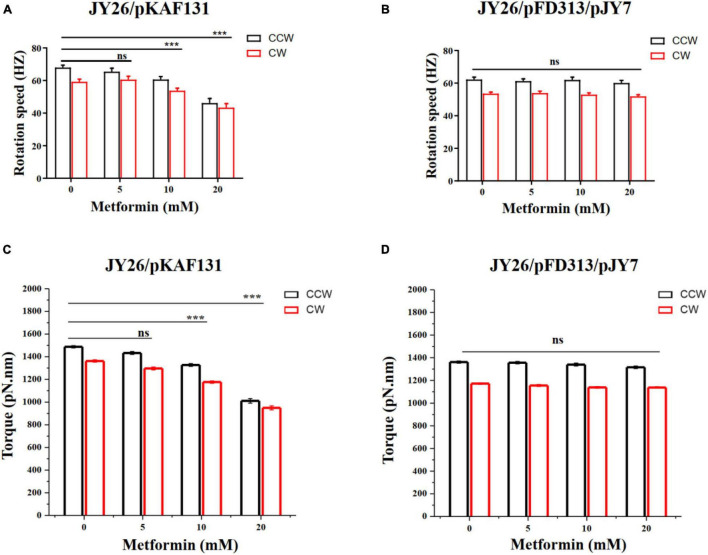
The rotation speed and torque of the flagellar motor of *E. coli* cultured with various concentrations of metformin. **(A)** The CCW (black) and CW (red) rotation speed of the flagellar motor of the cells that normally express MotA and MotB. The numbers of the motors measured at each point were 43, 38, 48, and 51, respectively. **(B)** The CCW (black) and CW (red) rotation speed of the flagellar motor of the cells that overexpress MotA and MotB. The numbers of the motors measured at each point were 46, 52, 52, and 46, respectively. **(C)** The flagellar motor torque of the cells in **(A)**. **(D)** The flagellar motor torque of the cells in **(B)**. The bars and errors are MEAN and SEM. ***p < 0.001.

To investigate the effect of metformin on the flagellar motor rotation of *E. coli*, the strain JY26 transformed with the plasmid pKAF131 that constitutively expresses the sticky filament FliC*^st^* which was used in this experiment. The rotation of 180 motors on different cells cultured with the gradient (0, 5, 10, and 20 mM) of metformin was recorded, with each motor observed for about 200 s. The motor rotation speed was analyzed using custom scripts in MATLAB (MathWorks) (Wang et al., 2014, 2017). The average motor rotation speed of the cells in the CCW and CW directions is shown in Figure 3A. Both the CCW motor rotation speed and CW motor rotation speed were decreased with the concentration of metformin. The motor rotation speed of JY26 cultured with 20 mM metformin decreased dramatically, reducing by 32.1 and 26.8% compared to the control in CCW direction and CW direction. Hence, the metformin inhibited the motor rotation speed of *E. coli*.

**FIGURE 4:**
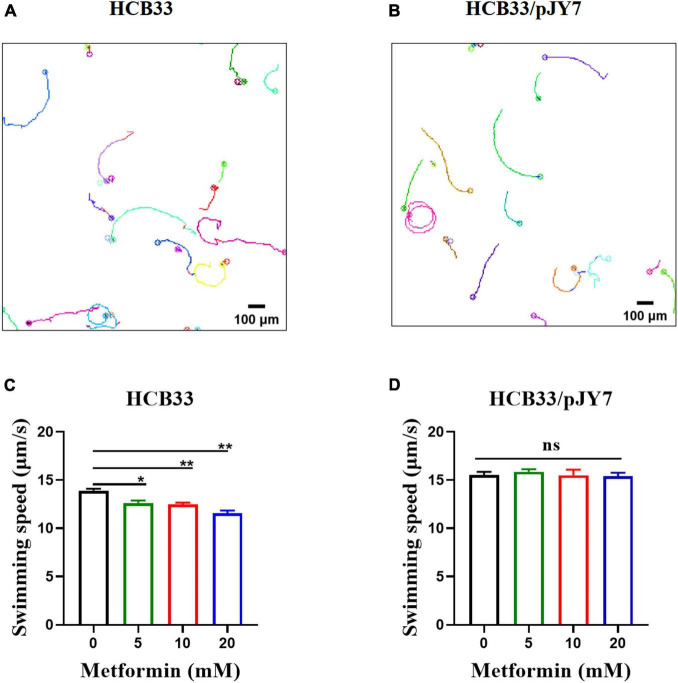
Swimming of the cells cultured with various concentrations of metformin in a 2D homogeneous aqueous environment. **(A)** Trajectories of the cells that normally express MotA and MotB. **(B)** Trajectories of the cells that overexpress MotA and MotB. **(C)** The average swimming speed of the cells that normally express MotA and MotB cultured in T-broth with different concentrations of metformin. **(D)** The average swimming speed of the cells that overexpress MotA and MotB cultured in T-broth with different concentrations of metformin. *p < 0.05; **p < 0.01.

We further measured the swimming motility of individual cells by tracking the trajectories of bacteria cultured with the gradient of metformin near the surface in a 2D homogeneous aqueous environment. Representative trajectories of *E. coli* are shown in Figures 4A,B. To quantify the observations, we calculated the instantaneous swimming speed and tumble frequency from the tracking data (Supplementary Figure 2). The bacteria were cultured in the gradient of metformin (0, 5, 10, and 20 mM). Trajectories longer than 5 s were used for further analysis. For the wild-type strain HCB33, a total of 765, 850, 969, and 659 trajectories with total times of 119, 114, 110, and 103 min were obtained corresponding to the gradient of metformin, respectively. Then, we calculated the average swimming speed of the bacteria cultured with different concentrations of metformin. The results showed that bacteria cultured with metformin swim slower in an aqueous environment, especially for 20 mM metformin (Figure 4C). Compared with the control, the average swimming speed of bacteria cultured with 20 mM metformin decreased by 16.87%. The result was consistent with the consequence above the flagellar motor rotation which was inhibited by metformin.

**FIGURE 5:**
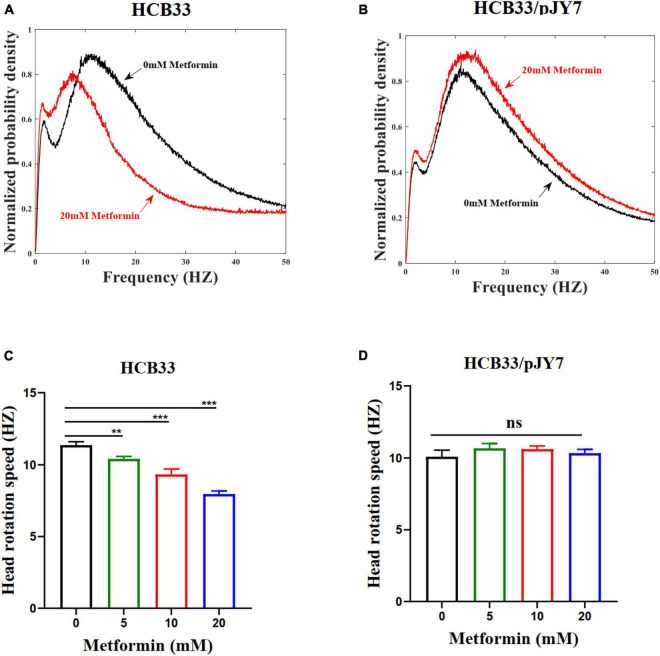
The head rotation speed of the flagellar motor of *E. coli* cultured with various concentrations of metformin. **(A)** The cells that normally express MotA and MotB cultured in T-broth with 0 mM metformin (black line) and cultured in T-broth with 20 mM metformin (red line). **(B)** The cells that overexpress MotA and MotB cultured in T-broth with 0 mM metformin (black line) and cultured in T-broth with 20 mM metformin (red line). **(C)** The average head rotation speed of the cells that normally express MotA and MotB was 11.37 ± 0.23 Hz, 10.41 ± 0.17 Hz, 9.33 ± 0.37 Hz, and 7.96 ± 0.21 Hz corresponding to the concentrations of metformin. **(D)** The average head rotation speed of the cells that overexpress MotA and MotB was 10.09 ± 0.47 Hz, 10.67 ± 0.31 Hz, 10.62 ± 0.22 Hz, and 10.34 ± 0.24 Hz corresponding to the concentrations of metformin. The bars and errors are MEAN and SEM. **p < 0.01; ***p < 0.001.

To verify whether metformin had an inhibition to the swimming motility of the cell population, we observed the average angular rotation speed of the cell head of the population by a high-throughput dark-field flicker microscopy (Martinez et al., 2014). The wild-type strain HCB33 was used in this experiment. The head rotation speed for the wild-type strain cultured with the gradient of metformin for a large population of cells was recorded (Figure 5A). The data collected in various concentrations of metformin were 29, 29, 29, and 30, respectively. The head rotation speed was distinct with the metformin concentration with cells cultured in 20 mM metformin decreased dramatically, reducing by 29.6% compared to the control of 0 mM metformin (Figure 5C). The results clearly demonstrated that the swimming motility of the cell population was inhibited by metformin. We also examined the swimming motility of the strain HCB33 in a semi-soft agar plate with the gradient of metformin. Undoubtedly, the swimming diameter was shortened with the concentration of metformin (Supplementary Figure 3A).

**FIGURE 6:**
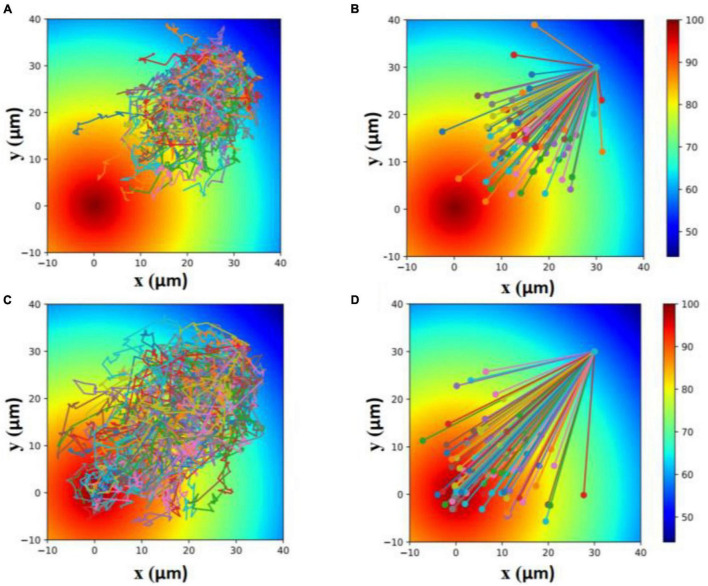
Simulation of the locomotion of cells with poor motility **(A,B)** and cells with normal motility **(C,D)** toward the attractant within 100 s. Each line in **(A,C)** indicates a 100-s trajectory with the start position to the end position. Each line in **(B,D)** indicates a 100-s straight line from the start position to the end position.

The inhibition of the flagellar motility of *E. coli* by metformin reduced the locomotion efficiency of cells. To show that, we made a simulation of the locomotion of cells with normal motility (cells cultured without metformin) and cells with poor motility (cells cultured with metformin) toward the attractant. As shown in Figure 6, cells with poor motility could only explore a narrower region toward the attractant within the same period.

Taken together, the results above implied that the flagellar motility of *E. coli* was inhibited by metformin. Further simulation suggested that the inhibition of flagellar motility led to the decrease in locomotion efficiency of cells.

### The Motility Inhibition Is Due to Reduced Torque of the Flagellar Motor

We hypothesized that the motility inhibition of metformin to cells might be caused by the difference of the motor torque (Xing et al., 2006; Wang et al., 2017). To test whether the motility inhibition was caused by the reduction of the motor torque, we used the strain JY26 transformed with plasmids pJY7 and pFD313. The plasmid pJY7 expresses wild-type MotA and MotB under the control of an arabinose-inducible promoter, while the plasmid pFD313 expresses the sticky filament FliC*^st^* constitutively (Wang et al., 2014). MotA and MotB were overexpressed in JY26, with the thought that with excess MotA and MotB, it would be likely to restore to the wild-type level for the stator proteins binding to the flagellar motor of the cells cultured with various concentrations of metformin. The rotations of 196 motors on different cells cultured with various concentrations of metformin were recorded, with each motor observed for about 200 s. The motor rotation speed in the CCW and CW directions showed no significant difference (Figure 3B). We further calculated the motor torque of cells normally expressing or overexpressing MotA and MotB. The results of flagellar motor rotation speed and the motor torque of cells that normally express or overexpress MotA and MotB are clearly illustrated in Table 2. The torques of cells normally expressing MotA and MotB were reduced with the concentration of metformin (Figure 3C), while the torques of cells overexpressing MotA and MotB were unchanged with the concentration of metformin (Figure 3D).

**TABLE 2:** The average CCW and CW rotation speed and torque of the flagellar motor for cells that normally express and overexpress MotA and MotB.

Strains	Metformin (mM)	CCW (HZ)	CW (HZ)	CCW torque (pN.nm)	CW torque (pN.nm)
JY26/pKAF131	0	67.95 ± 1.51	59.21 ± 1.64	1487.84	1326.77
	5	65.45 ± 2.16	60.59 ± 2.05	1433.13	1296.53
	10	60.63 ± 1.84	53.74 ± 1.55	1327.61	1176.71
	20	46.16 ± 2.86	43.34 ± 2.55	1010.77	949.03
JY26/pFD313/pJY7	0	65.45 ± 2.16	60.59 ± 2.05	1433.13	1172.47
	5	61.21 ± 1.46	53.91 ± 1.23	1356.86	1180.22
	10	61.97 ± 1.75	52.97 ± 1.09	1340.23	1159.84
	20	60.11 ± 1.65	51.92 ± 1.01	1316.03	1136.82

We further measured the swimming motility of individual cell and cell populations, along with swimming in semi-soft agar plates of the cells overexpressing MotA and MotB. The results of the average swimming speed of cells that normally express or overexpress MotA and MotB are clearly illustrated in Table 3. The average swimming speed of individual cells that overexpress MotA and MotB was restored (Figure 4D). The bacteria were cultured in the gradient of metformin (0, 5, 10, and 20 mM). Trajectories longer than 5 s were used for further analysis. For the strain HCB33 with plasmid pJY7, a total of 805, 1,002, 780, and 888 trajectories with total times of 103, 129, 97, and 111 min were obtained corresponding to the gradient of metformin, respectively. The results of the cell population that overexpresses MotA and MotB revealed that the head rotation speed was also restored (Figures 5B,D). The data collected in various concentrations of metformin were 23, 23, 23, and 26, respectively. The results of swimming in semi-soft agar plates of the cells overexpressing MotA and MotB showed that the swimming motility was restored as well (Supplementary Figure 3B).

**TABLE 3:** Mean swimming speeds for cells that normally express and overexpress MotA and MotB.

Metformin (mM)	Strains	
	HCB33 Swimming speed (μ m/s)	HCB33/pJY7 Swimming speed (μ m/s)
0	13.93 ± 0.26	15.51 ± 0.44
5	12.64 ± 0.36	15.87 ± 0.38
10	12.48 ± 0.29	15.51 ± 0.82
20	11.58 ± 0.42	15.29 ± 0.54

Therefore, the results above clearly demonstrated that cells overexpressing MotA and MotB restored the flagellar motility that governed the flagellar motor rotation and the swimming of cells in spite of being cultured with metformin. As the flagellar motility of the bacteria depended strongly on the flagellar motor torque, the consequences suggested that the motility inhibition of metformin to cells was due to the reduction of motor torque.

## Discussion

Recent studies have shown that metformin plays an important role in delaying aging, improving life span, and improving glucose metabolic diseases, which involves the cooperation of intestinal microbiota (Bailey et al., 2008; Cabreiro et al., 2013; Maratos-Flier, 2013; De Haes et al., 2014; Forslund et al., 2015; Wu et al., 2017; Fang et al., 2018). For example, metformin slowed the aging of *C. elegans* by altering the metabolism of folate and methionine in *E. coli*; another study showed that metformin can affect the distribution of intestinal microbiota and regulate glucose metabolism of the host by regulating intestinal microbiota (Cabreiro et al., 2013; Bauer et al., 2018). Metformin could inhibit the growth of bacteria such as *E. coli* and *H. pylori* (Cabreiro et al., 2013; Courtois et al., 2018). Metformin also exhibited the potential effect on tetracycline antibiotics, particularly doxycycline and minocycline, against multidrug resistance *S. aureus*, *E. faecalis*, *E. coli*, and *S. enteritidis* (Liu Y. et al., 2020). It is clear that metformin is closely related to intestinal microbiota and metabolism. Although many studies have been conducted on metformin, the effect of metformin on the physiological behavior of bacteria such as chemotaxis and motility remained unclear. In this study, we report a new property of metformin that alters the behavior of chemotaxis and motility of *E. coli*.

In summary, we measured the chemotactic response curve of wild-type cells for chemotaxis and cells lacking tsr chemoreceptors to metformin, finding that metformin was sensed by tsr as a repellent to *E. coli*. Furthermore, we measured the chemotactic response of wild-type cells for chemotaxis cultured in metformin to glucose and MeAsp. The results indicate that metformin prolonged the chemotactic recovery time of *E. coli* to the attractant. Previous findings revealed that the chemotaxis adaptation of *E. coli* to the attractant was ultrasensitive, wide-ranged, and robust (Alon et al., 1999; Mello and Tu, 2007; Liu G. et al., 2020). In this study, we showed that the chemotactic response of wild-type cells for chemotaxis cultured in metformin to the attractant was different from cells cultured without metformin, in spite of the overall tendency of chemotactic response being similar. Hence, our results indicated that metformin altered the chemotaxis of *E. coli* by prolonging the recovery time of *E. coli* chemotactic response to attractant.

Moreover, we systematically studied the effect of metformin on bacterial flagellar motility from the level of the single flagellar motor to the individual cell and cell population. For the first time, our results illustrated that metformin inhibits the motility of cells: inhibition of the flagellar motor rotation, inhibition of individual cell swimming motility, and inhibition of swimming motility of the cell population. A previous study revealed that the rotation speed of the flagellar motor of *E. coli* varied linearly with PMF (Gabel and Berg, 2003). The rotation speed of the flagellar motor decreased when PMF was reduced (Wang et al., 2017). Recent work demonstrated that metformin disrupted the transmembrane potential and dramatically undermined the functions of PMF-driven efflux pump in bacteria (Liu Y. et al., 2020). Thus, it seems plausible that the reduction of PMF by metformin resulted in the inhibition of the flagellar motility. Since the torque was assessed by motor speed and external load which tuned the motility of cells, our results indicated that the motor torque was reduced by metformin. We further performed the experiments with cells overexpressing MotA and MotB to elevate the torque, finding that the inhibition to the motility of cells by metformin was vanished. Previous studies showed that the motor was a dynamic structure with Mot proteins binding on and off it (Leake et al., 2006; Reid et al., 2006). Under the same external load, the more there are Mot proteins binding on the motor, the higher the motor rotation speed (thus the higher torque generated by the motor). Our results suggested that the motility inhibition by metformin was due to reduced torque of the flagellar motor.

Cells of *E. coli* swim up gradients of chemical attractants in the aquatic environment by modulating their flagellar motor rotation direction between CCW and CW, which determines the cell run or tumble. The motility determined by rotating bacterial flagellar motor and flagellum was also involved in the searching of nutrients by bacteria. Cells with higher swimming speed spent less time to migrate toward nutrients compared to cells with lower motility. Our study showed that the motility of cells cultured with metformin was reduced in comparison with the control under the same external condition. Metformin, as a chemical repellent to *E. coli*, reduced the locomotion efficiency of cells. The simulation results clearly demonstrated that cells cultured with metformin could only explore a narrower region toward the attractant within the same period due to the reduction of flagellar motility.

Overall, our findings reveal the new property of metformin that alters the chemotaxis and motility of *E. coli* for the first time. We expect to find similar examples of the behavior alterations in chemotaxis and motility of other microorganisms affected by metformin. Considering that chemotaxis and motility are vital to bacteria such as *E. coli*, which could affect a variety of key physiological functions of bacteria, our discovery could provide a new and potential perspective to study the effect of metformin on bacteria.

## Data Availability Statement

The original contributions presented in the study are included in the article/Supplementary Material, further inquiries can be directed to the corresponding author/s.

## Author Contributions

FW planned the work. YY and PJ performed the measurements. FW and YY wrote the manuscript with inputs from other authors. All authors contributed to the article and approved the submitted version.

## Conflict of Interest

The authors declare that the research was conducted in the absence of any commercial or financial relationships that could be construed as a potential conflict of interest.

## Publisher’s Note

All claims expressed in this article are solely those of the authors and do not necessarily represent those of their affiliated organizations, or those of the publisher, the editors and the reviewers. Any product that may be evaluated in this article, or claim that may be made by its manufacturer, is not guaranteed or endorsed by the publisher.
