# Supplementary material for: Metformin Alters the Chemotaxis and Flagellar Motility of Escherichia coli
Source: Front Microbiol. 2022 Jan 11;12:792406. doi: 10.3389/fmicb.2021.792406 (PMC8787215; doi:10.3389/fmicb.2021.792406)
Supplement: Supplementary file 1 [file Data_Sheet_1.docx]

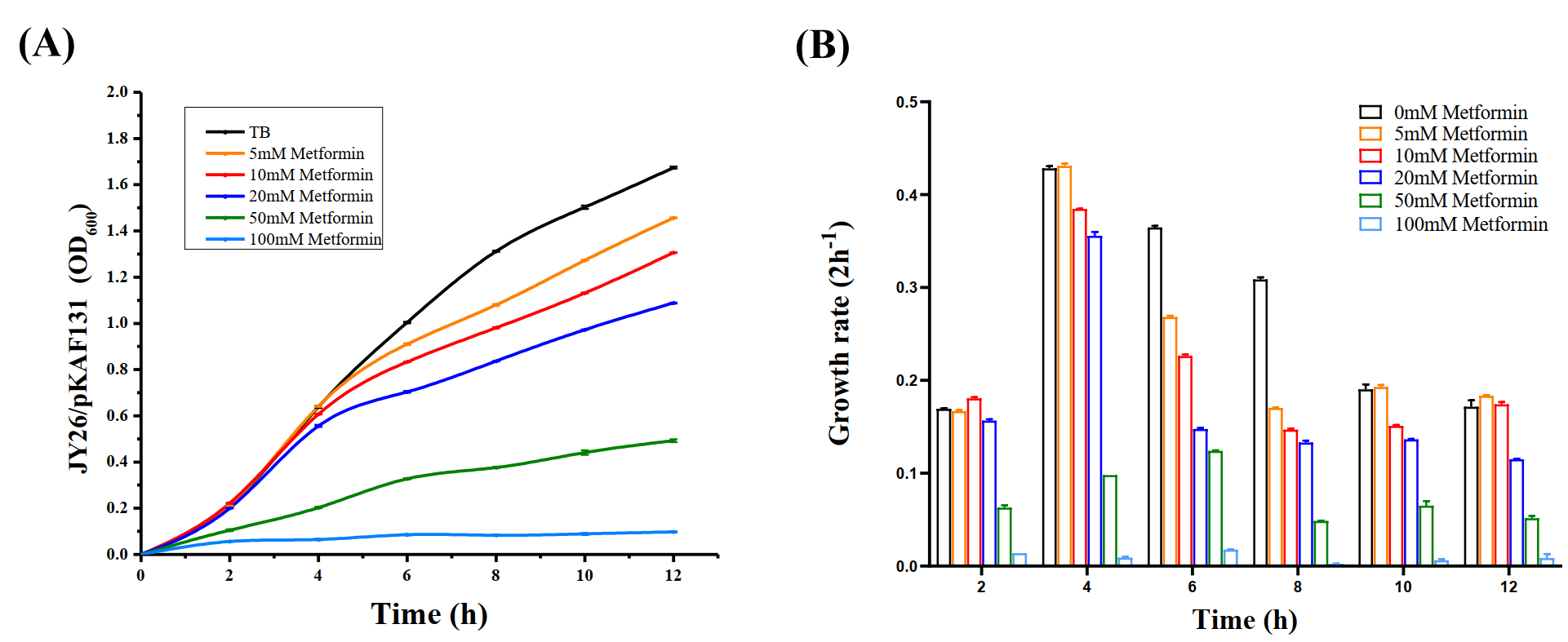


Fig S1. The influence of metformin on *E. coli* growth. (A) The growth curves of the strain JY26/pKAF131 cultured with various concentrations of metformin. The optical density (OD_600_) was measured every two hours. (B) The growth rate of the JY26/pKAF131 cultured with various concentrations of metformin.


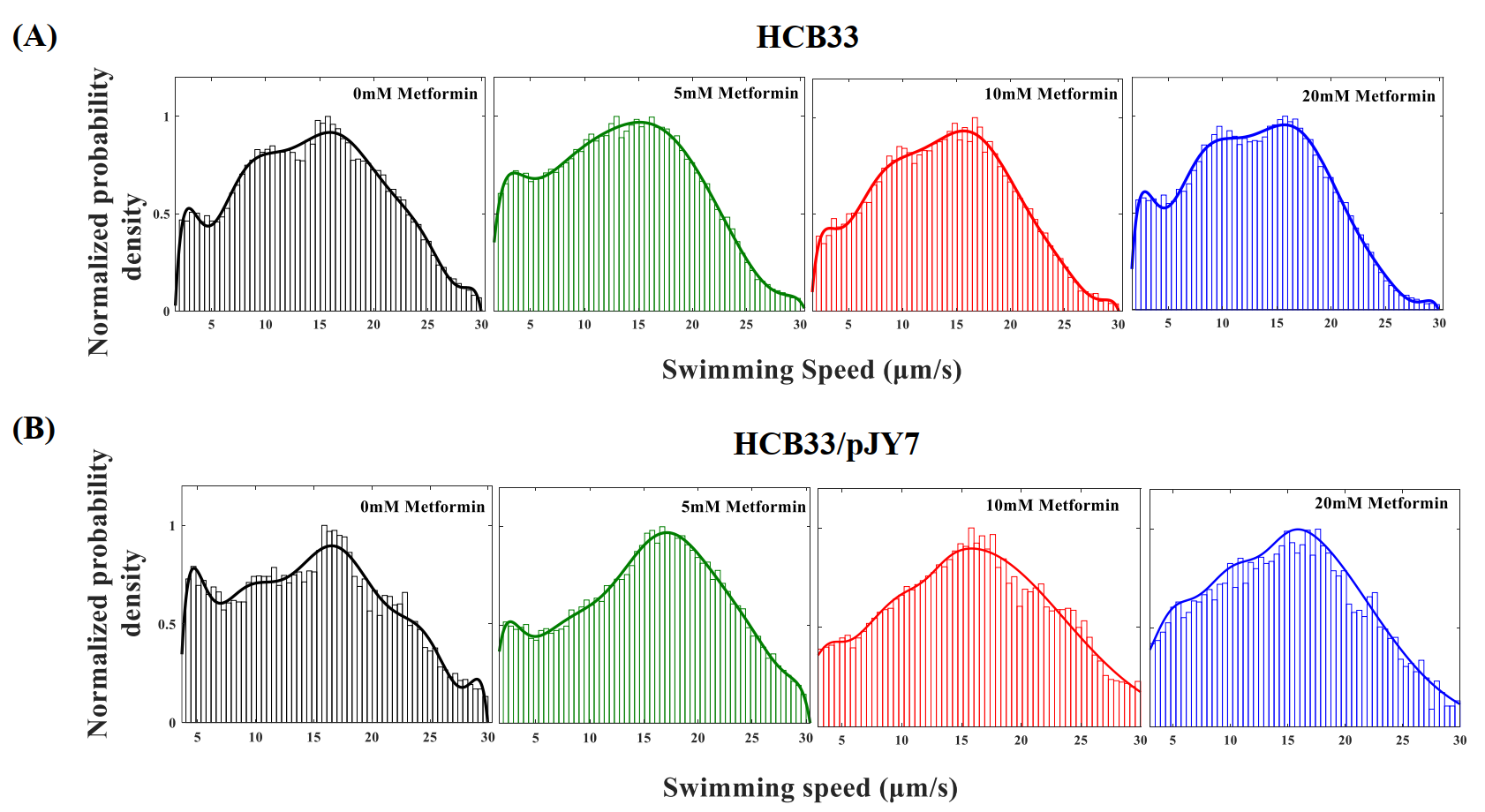


Fig S2. The swimming speed distribution of HCB33 (A) and HCB33/pJY7 (B) cells in a 2D aqueous environment.


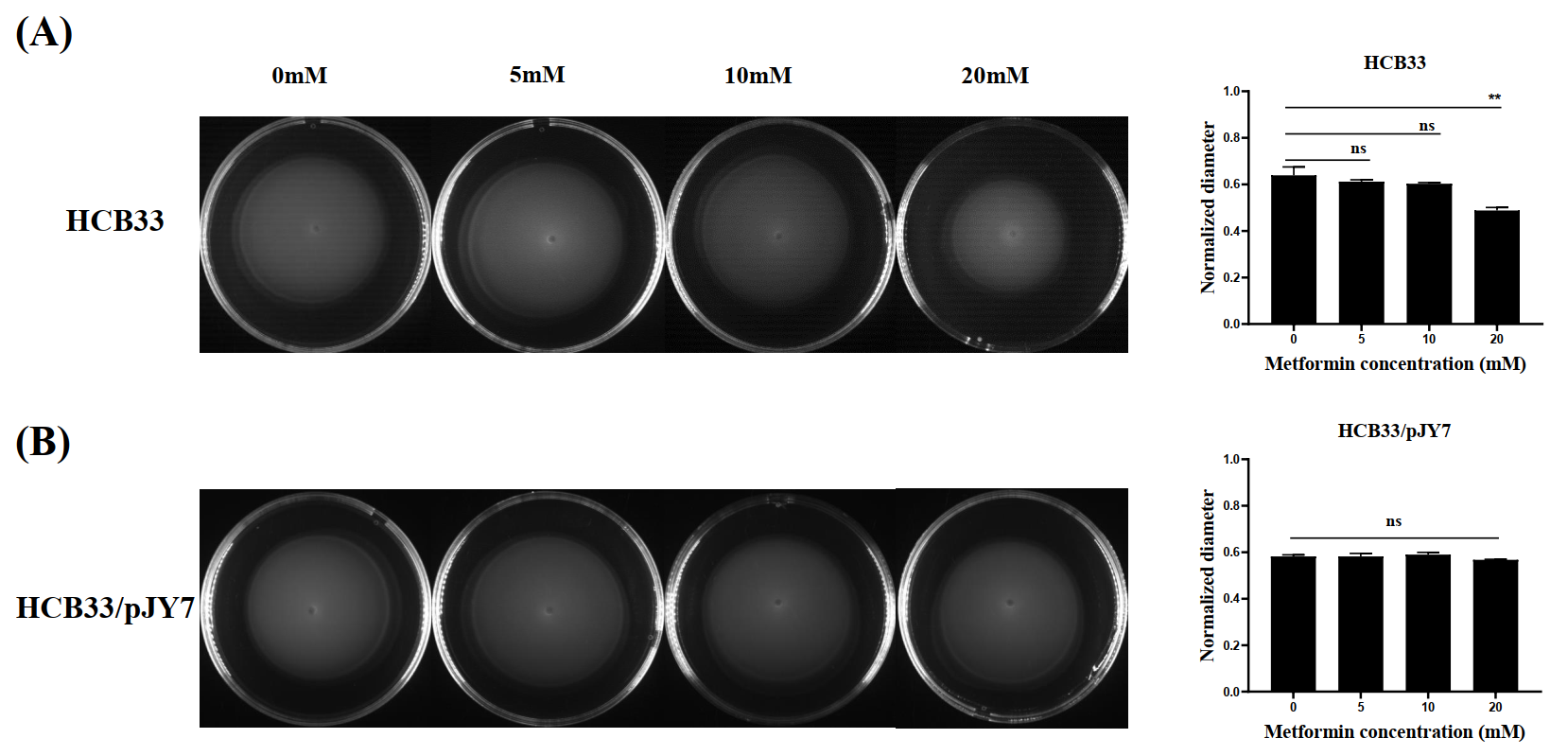


Fig S3. Swimming motility of the wild-type strain HCB33 (A) and the strain HCB33 /pJY7 (B) under various concentrations (0mM, 5mM, 10mM and 20mM) of metformin on soft-agar plates. Plates were imaged after cells were spotted in the center and incubated for 12 hours at 37°C. Culture diameters were normalized to the diameter of the petri dishes. The bars and errors are MEAN and SEM. *p < 0.05; **p < 0.01; ***p < 0.001.
